# Serum soluble Fas ligand is a severity and mortality prognostic marker for COVID-19 patients

**DOI:** 10.3389/fimmu.2022.947401

**Published:** 2022-08-31

**Authors:** Kiarash Saleki, Moein Shirzad, Mostafa Javanian, Sheyda Mohammadkhani, Mohammad Hossein Alijani, Niloufarsadat Miri, Morteza Oladnabi, Abbas Azadmehr

**Affiliations:** ^1^ Cellular and Molecular Biology Research Center, Health Research Institute, Babol University of Medical Sciences, Babol, Iran; ^2^ Student Research Committee, Babol University of Medical Sciences, Babol, Iran; ^3^ USERN Office, Babol University of Medical Sciences, Babol, Iran; ^4^ National Elite Foundation, Mazandaran Province Branch, Mazandaran, Iran; ^5^ Infectious Diseases and Tropical Medicine Research Center, Health Research Institute, Babol University of Medical Sciences, Babol, Iran; ^6^ Ischemic Disorders Research Center, Golestan University of Medical Sciences, Gorgan, Iran; ^7^ Immunology Department, Babol University of Medical Sciences, Babol, Iran

**Keywords:** COVID-19, hyperinflammation, Fas, viral Immunology, immunoinformatics

## Abstract

Finding cytokine storm initiator factors associated with uncontrolled inflammatory immune response is necessary in COVID-19 patients. The aim was the identification of Fas/Fas Ligand (FasL) role in lung involvement and mortality of COVID-19 patients. In this case-control study, mild (outpatient), moderate (hospitalized), and severe (ICU) COVID-19 patients and healthy subjects were investigated. RNA isolated from PBMCs for cDNA synthesis and expression of mFas/mFasL mRNA was evaluated by RT-PCR. Serum sFas/sFasL protein by ELISA and severity of lung involvement by CT-scan were evaluated. Also, we docked Fas and FasL *via* Bioinformatics software (*in silico*) to predict the best-fit Fas/FasL complex and performed molecular dynamics simulation (MDS) in hyponatremia and fever (COVID-19 patients), and healthy conditions. mFasL expression was increased in moderate and severe COVID-19 patients compared to the control group. Moreover, mFas expression showed an inverse correlation with myalgia symptom in COVID-19 patients. Elevation of sFasL protein in serum was associated with reduced lung injury and mortality. Bioinformatics analysis confirmed that blood profile alterations of COVID-19 patients, such as fever and hyponatremia could affect Fas/FasL complex interactions. Our translational findings showed that decreased sFasL is associated with lung involvement; severity and mortality in COVID-19 patients. We think that sFasL is a mediator of neutrophilia and lymphopenia in COVID-19. However, additional investigation is suggested. This is the first report describing that the serum sFasL protein is a severity and mortality prognostic marker for the clinical management of COVID-19 patients.

## Introduction

Coronaviruses (CoVs) created two pandemics in the world by the Severe Acute Respiratory Syndrome (SARS)-CoV (1) and Middle-East respiratory syndrome (MERS)-CoV (2) variants, respectively. Recently, SARS-CoV2 initiated a third pandemic called Coronavirus disease-2019 (COVID-19). This pandemic is a major cause of mortality in the world (3).

On the other hand, COVID-19 is commonly accompanied by a harmful inflammatory response, and overactivation of neutrophils which leads to the formation of Neutrophil Extracellular Traps (NETs), sepsis, and cytokine storm (4, 5). Cytokine storm is one of immunopathological reactions in COVID-19 patients, which is an aggressive inflammatory response with excessive release of cytokines and elements, including interleukin (IL)-2, IL-6, IL-7, IL-10, tumor necrosis factor (TNF), granulocyte colony-stimulating factor (G-CSF), monocyte chemoattractant protein-1 (MCP1; or CCL2), macrophage inflammatory protein 1 alpha (MIP1α; or CCL3), CXC-chemokine ligand 10 (CXCL10), C-reactive protein (CRP), ferritin, and D-dimers (6–8). In addition to cytokine storm, other inflammatory factors such as sFasL increase disease severity and inflammation in COVID-19 patients (9).

We recently proposed a novel triangle in COVID-19, comprising viral infection, cytokine storm, and multi-system damage, such as respiratory and CNS. We described how this triangle dysregulates immune response through Fas/Fas Ligand (FasL) in COVID-19 patients (10–13). Soluble FasL (sFasL) is produced by the cleavage of membrane-attached FasL (mFasL) *via* a zinc-regulated matrix metalloprotease (MMP). mFas/mFasL and sFas/sFasL interactions could induce hyperinflammation and recruit immune cells playing a possible role in the multi-system injury in COVID-19 patients (12).

A new study showed that raised serum sFasL in severe burn is associated with pro-inflammatory cytokine production, organ injury, lymphopenia, and predicts mortality (14). André et al. showed that T-lymphocytes death in COVID-19 patients could be mediated by sFasL and CXCL10, that leads to hyperinflammation in this condition (15). Moreover, mFasL and sFasL lead the inflammaging processes, that are related to viral lung disorders (16). Kessel et al. explained that sFasL is a discriminating factor that can lead to a dysregulated immune response and amplify inflammation in COVID-19 patients (17). Finally, evidence suggests that sFasL may be distinctly implicated in COVID-19 as an important factor in the inflammatory response and contribute to mortality.

In this study, we evaluated mFas/mFasL expression by RT-PCR, sFas/sFasL serum level by ELISA, severity of disease by computed tomography (CT)-scan in COVID-19 patients and utilized *in silico* (Bioinformatics) software to study Fas/FasL interactions.

## Methods

### Sample selection and data collection

This study enrolled 120 subjects in four groups in 2020-2021; including healthy controls, COVID-19 out-patients (mild), hospitalized COVID-19 (moderate), and ICU COVID-19 (severe). The inclusion criteria for healthy controls were I; no history of any major condition that is associated with hyperinflammation, such as obesity (BMI>30), diabetes, severe cardiovascular, renal, cerebrovascular, or autoimmune disorders, II; not being vaccinated for COVID-19, III; not being infected with or recovering from a recent COVID-19 infection. COVID-19 patients were included without age limitation. Demographic information, clinical symptoms of COVID-19, and past medical history were obtained for COVID-19 patients. Additional laboratory data, such as CBC, C-reactive protein (CRP), ESR, electrolytes, as well as vital signs and O2 saturations were collected for hospitalized and ICU COVID-19 patients. COVID-19 diagnosis was confirmed by serological rapid IgM assay and real-time polymerase chain reaction (RT-PCR). Furthermore, hospitalization or admission of patients to ICU was carried out according to different clinical parameters, including SpO_2_, lung computed tomography (CT)-scan, heart rate, and various blood parameters such as LDH, CPK, Ferritin, Troponin, C-reactive protein (CRP) and interleukin-6 (IL-6), similar to criteria provided by the National Institutes of Health (NIH) (https://www.covid19treatmentguidelines.nih.gov) and National Guidelines of the Diagnosis and Treatment Committee for COVID-19. Also, COVID-19 mortality was followed by the medical record center of referred hospital as well as *via* phone calls (the patients him/herself or through patients’ family members).

### PBMCs isolation, RNA extraction, and cDNA synthesis

Venous whole blood specimens (5 cc) were transferred to collection tubes with EDTA. Peripheral blood mononuclear cells (PBMCs) were isolated *via* lymphocyte separation media. The blood samples by Ficoll as the lymphocyte separation media, were centrifuged at 800 ×g for 20 min. Then, the PBMCs were washed twice by phosphate buffered saline (PBS), at pH 7.4. RNA was extracted from PBMCs by Total RNA Extraction Kit (Yekta tajhiz Azma, Tehran, Iran) based on the manufacturer’s instructions. The Easy™ cDNA Reverse Transcription kit (ParsTous, Iran) was used to perform reverse transcription of total RNA into cDNA. Briefly, the template RNA (total RNA or Poly (A) mRNA) and other kit components in RNase-free tube were mixed. Next, the mixture was rapidly vortexed and then, incubation was performed for 10 min at 25°C and 1 hour at 47°C, respectively. The reaction was stopped by heating at 85°C for 5 minutes. At the end, cDNA products were stored at -80°C.

### Serum isolation

Venous whole blood samples from COVID-19 cases and normal participants were obtained in Falcon collection tubes without EDTA. The blood specimens were then centrifuged at 1000 xg for 10 min to remove the clots. The supernatant was collected and stored at -80°C and used for enzyme-linked immunosorbent assay (ELISA) experiment.

### Expression analysis of Fas and FasL by real-time PCR

Primer sequences that were used are mentioned in **Table 1** (18–20). The used sequences were oligonucleotide primers for the recognition of Fas and FasL. NCBI blast was also used to check the primer against human genome. Glyceraldehyde 3-phosphate dehydrogenase (GAPDH) was selected as reference housekeeping gene, with same primers as our recent previous study (20). These sequences were re-analyzed for suitability by Oligo7. In order to assess relative mRNA expression RT-PCR was performed.

**Table 1:** Primer sequences.

Gene	Direction	Sequence 5’ to 3’	Reference
**Fas** **Fas**	Forward Reverse	TGAAGGACATGGCTTAGAAGTG GGTGCAAGGGTCACAGTGTT	(18)
**FasL** **FasL**	Forward Reverse	GCAGCCCTTCAATTACCCAT CAGAGGTTGGACAGGGAAGAA	(19)
**GAPDH** **GAPDH**	Forward Reverse	ACAGTCAGCCGCATCTTC CTCCGACCTTCACCTTCC	(20)

After amplifying cDNAs using PCR with SYBR Green qPCR Master Mix 2X (ParsTous Biotechnology, Mashhad, Iran), we performed real-time PCR test using Applied Biosystems 7300 real-time thermocycler (Applied Biosystems, MA, USA).

We assessed the expression level of Fas and FasL mRNA in mild, moderate, and severe COVID-19 cases, and control subjects, as expression ratios normalized to the expression of the house keeping gene, GAPDH.

### Analysis of Fas and FasL protein concentrations by ELISA

Serum of patients was used to measure Fas and FasL proteins. Their levels were quantified for 88 subjects by the BIOTECH ELISA kit for human Fas and FasL (SHANGHAICRYSTAL DAY BIOTECH CO., Shanghai, China), respectively. Concentrations were measured at 450 nm by Synergy HTX multi-mode microplate reader (BioTek Instruments, Winooski, VT, USA) according to the color shade of each plate. We assessed the concentration of Fas and FasL proteins in mild, moderate and severe COVID-19 patients as well as the control group.

### Correlation analysis of lung injury CT scan and sFas/sFasL protein levels

Lung has two right and lobes, that includes upper, middle, and lower lobes. Each lobe is about one-sixth of lung area, and percent of involvement is determined by this method in our department. We collected the lung CT-scan results that were available for COVID-19 patients. Correlation analysis was performed between lung injury and sFas/sFasL protein levels that were detected by ELISA.

### The *in silico* molecular dynamics study of Fas/FasL interaction in blood

Immunoinformatics is carried using various software that extend our knowledge of molecular findings by facilitating processes such as simulating complex reactions and screening compounds for experimental evaluation of drugs (21). Therefore, we evaluated Fas/FasL interactions *in silico* by simulating the blood profile condition of COVID-19 patients, such as fever and hyponatremia which is responsible for inflammation in this condition.

We modelled Fas and FasL by homology modelling and assessed structure quality. We used the best complex according to docking results. Molecular dynamics simulation (MDS) is a computational simulation method that is utilized for analysis of the physical movement of atoms and molecules. In MDS, a biological system is virtually set up and equilibrated, and is allowed a limited time to evolve. Then, the evolution of the system within the MDS trajectory can be analyzed. One of the most recognized and versatile programs for MDS is GRoningen MAchine for Chemical Simulations (GROMACS). This software package enables rapid and precise simulations (22).

We simulated the best docked Fas/FasL complex in blood *via* GROMACS. Visual Molecular Dynamics (VMD) (23) and UCSF chimera were employed to assess the simulation box during all MDS steps. Also, OPLS-AA force field was selected to produce topology of proteins (24, 25). Then, the Fas/FasL complex was centered in a triclinic box with edges separated by a minimum of 1 nm from the edges and also the simulation box enclosed the Fas/FasL complex. The box size was set to 6.2, 7.1, 5.2 nm. We used spce16 water for solvation. Moreover, we added Na^+^ and Cl^-^ ions to neutralize the system, and to reach a concentration of 150 mM similar to blood of healthy cases. Previous studies demonstrated that hyponatremia and fever are important pathophysiological findings in COVID-19 patients (26–31). Therefore, for COVID-19 groups; hyponatremia and fever, the simulation setting was modified to 130 mM/37°C and 150 Mm/38.4°C, respectively.

### Binding energy analysis of the contact area of the Fas/FasL complex

We selected protein binding energy prediction (PRODIGY) (https://wenmr.science.uu.nl/prodigy/), a novel tool that assesses the binding strength in biological molecules interactions. This algorithm has the ability to utilize alternative atomic radii and various nonpolar solvation modes (32). By this approach, we interconnected MDS with binding energy analysis for contact area of the Fas/FasL complex for all MDS groups. Negative energies were considered an indicator of favorable binding.

### Statistical analysis

Statistical Packages for Social Sciences (SPSS), (SPSS Inc., Chicago, USA) and GraphPad Prism version 8.4 (GraphPad Software Inc., La Jolla, CA, USA) were utilized to analyze RT-PCR and ELISA data. Moreover, data normality was evaluated *via* the Kolmogorov-Smirnov test for sample size (n ≥50) or Shapiro-Wilk for sample size (n ≤50) (33). ELISA data were assessed through one-way ANOVA with *post-hoc* Tukey analyses. Results were plotted as mean ± SEM. Moreover, *t* tests or non-parametric tests (e.g., Mann-Whitney) were used where appropriate. For all tests, threshold of significance was considered *p* = 0.05. For Bioinformatics analyses, suitable software packages and servers were used.

## Results

### Subject’s information and demographics

In this study, 120 individuals were enrolled, including mild (n=31), moderate (n=31), and severe (n=31) COVID-19 patients as well as healthy subjects (n=27), for whom gene expression analysis was performed. In the next step, the patients were followed-up for mortality and ELISA study. For follow-up subjects, age of participants was 48.59 ± 17.54, and 43 patients (48.86%) were male and 45 patients (51.14%) were female. As shown in detail in **Table 2**, clinical findings of COVID-19 patients included fever in 38 patients (57.57%), cough in 36 patients (54.55%), weakness in 31 patients (46.7%), myalgia in 30 patients (45.45%), nervous system symptoms in 22 patients (33.33%), along with GI symptoms such as nausea, vomiting, and diarrhea in 18 patients (27.27%). Inflammatory markers such as CRP and IL-6 as well as demographic information are provided in **Table 2**.

**Table 2:** Patient’s clinical characteristics.

Characteristic		COVID-19	Mild COVID-19	Moderate COVID-19	Severe COVID-19	Control group
**Gender (Male/Female); n (percentage)**		32/34 (48.5%/51.5%)	13/9 (59.1%/40.9%)	12/10 (54.4%/45.5%)	7/15 (31.8%/68.2%)	11/11 (50%/50%)
**Age (Mean)** **IL-6 (pg/mL); mean (SD)** **CRP (mg/L) mean (SD)**		52.37 57.49 (35.15) 51.20 (42.86)	39.55 16.40 (5.05) 14.97 (2.60)	57.22 67.06 (12.64) 65.42 (36.06)	60.36 89.01 (27.08) 73.21 (47.79)	37.68 5.15 (1.45) NA
**Symptoms**	Fever n (percentage)	38 (57.6%)	15 (68.2%)	14 (63.6%)	9 (40.9%)	0 (0%)
	Cough n (percentage)	26 (39.4%)	10 (45.5%)	7 (31.8%)	9 (40.9%)	0 (0%)
	Myalgia n (percentage)	30 (45.5%)	13 (59.1%)	9 (40.9%)	8 (36.4%)	0 (0%)
	Gastrointestinal n (percentage)	18 (27.3%)	5 (22.7%)	8 (36.4%)	5 (22.7%)	0 (0%)
	Neurological n (percentage)	19 (28.8%)	9 (40.9%)	3 (13.6%)	7 (31.8%)	0 (0%)

NA, Not Applicable.

### Expression of mFas and mfasL are attenuated in mild COVID-19 patients

**Figure 1:**
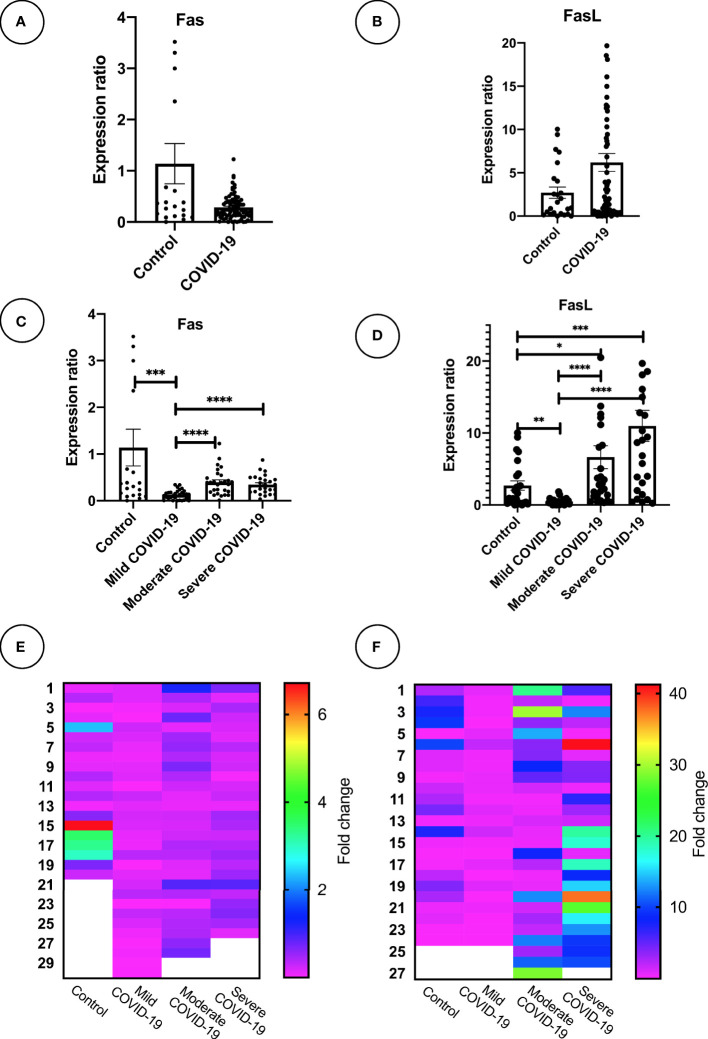
Fas and FasL mRNA expression in PBMC samples mRNA expression of Fas and FasL is shown for **(A, B)** COVID-19 (n = 93) and control group (n = 27). **(C, D)**, mild, moderate, and severe COVID-19 (n = 31 per group), and control group (n = 27). **(E, F)** Heatmaps graphically demonstrate expression level; *p<0.05, **p< 0.01, ***p<0.001, ****p< 0.0001. Outlier data which were abnormal were excluded for each gene expression analysis. *Outlier values are displayed as blanked in heatmaps. Higher values than graph range exceed the plot and may not be shown.

**Figure 2:**
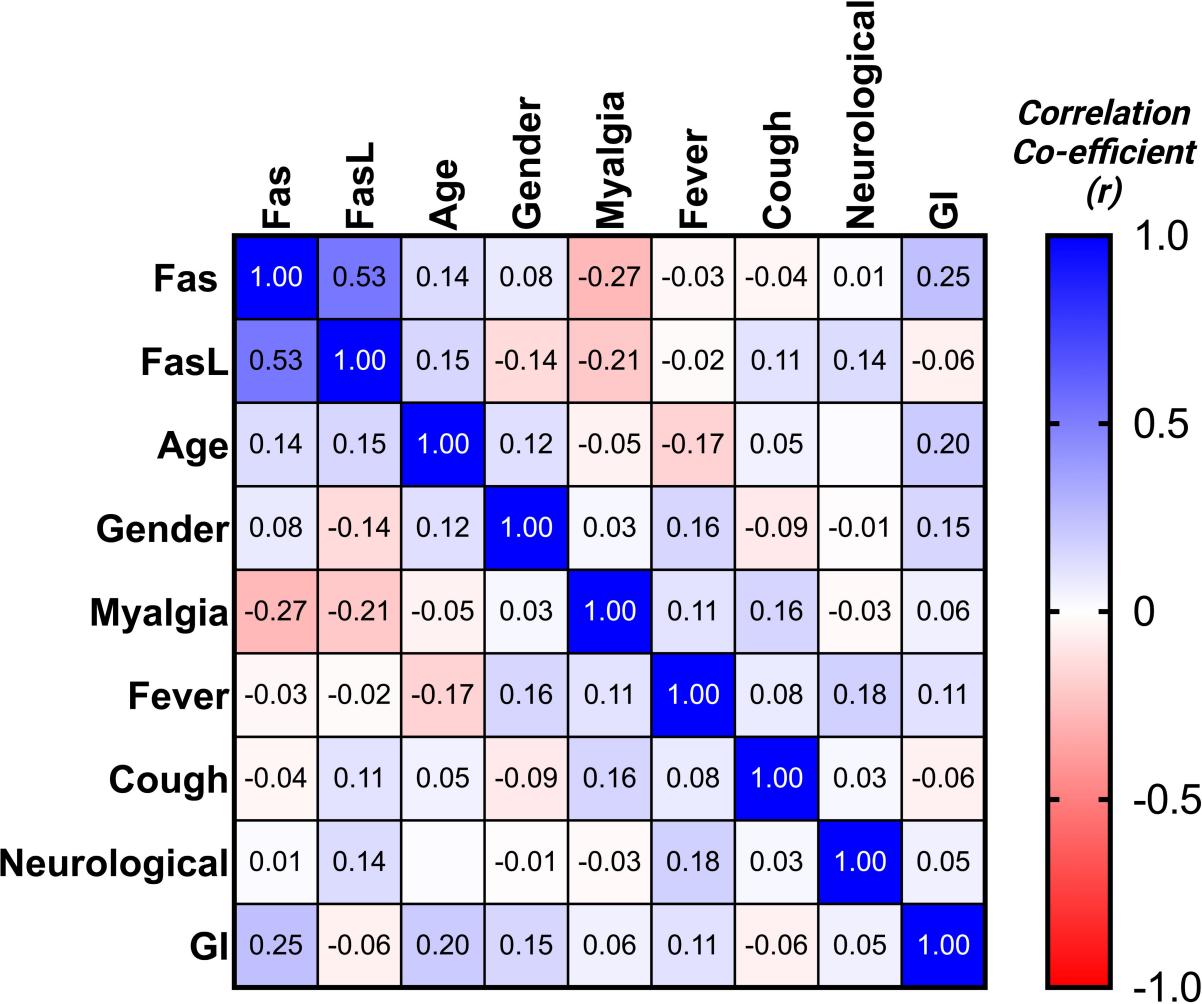
Correlation matrix for Fas/FasL and clinical disease properties.

Expression of mFas and mFasL mRNA was evaluated for all groups. COVID-19 patients in the moderate and severe COVID-19 groups showed significantly higher mFas expression compared to the mild COVID-19 group (p < 0.0001). Further, mild COVID-19 group had lower mFasL expression in comparison with the control group (p < 0.01) as well as moderate and severe COVID-19 groups (p < 0.0001). Overall, mFasL expression was higher in COVID-19 cases compared to the control group. mFasL expression was significantly elevated in moderate (p < 0.05) and severe (p < 0.001) COVID-19 groups compared to the control group. PCR results for mFas and mFasL are provided in **Figures 1A–F**. Also, correlation analysis of mFas/mFasL expression and clinical COVID-19 symptoms are provided in **Figure 2**.

### sFas and sFasL proteins are increased in mild COVID-19

**Figure 3:**
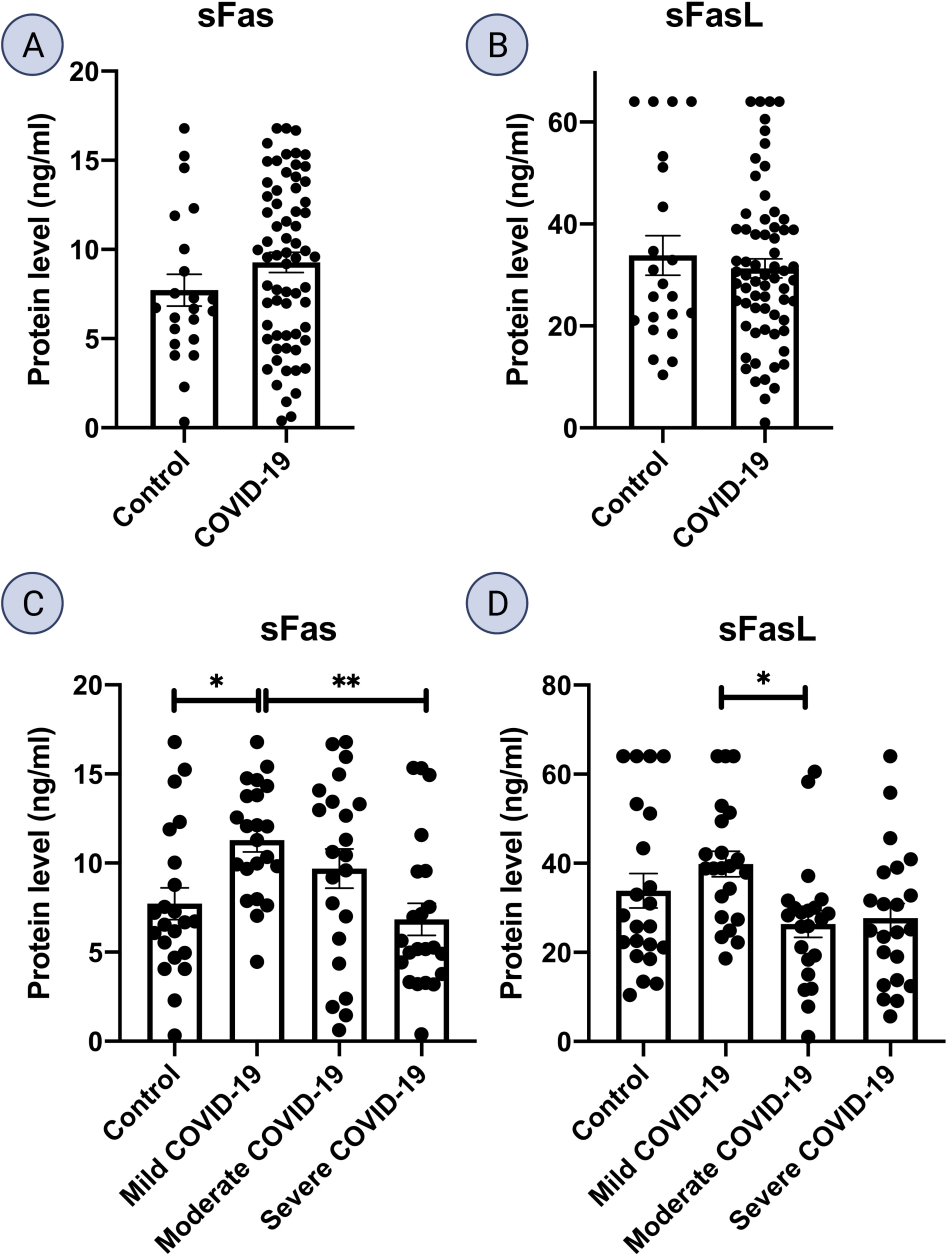
Fas and FasL protein level in serum samples. Serum levels of Fas and FasL is shown for **(A, B)** COVID-19 (n = 66) and control group (n = 22). **(C, D)**, mild, moderate, and severe COVID-19, and control group (n = 22 per group); *p<0.05, **p< 0.01, ***p<0.001, ****p< 0.0001.

ELISA analysis of serum samples from mild COVID-19 patients displayed an increase in both sFas and sFasL proteins compared to all other subgroups. sFas levels were significantly higher in mild COVID-19 group, compared to the control group (p < 0.05) and severe COVID-19 group (p < 0.01). sFasL levels were significantly higher in mild COVID-19 compared to moderate COVID-19 group (p < 0.05). Also, sFasL levels were lower in severe COVID-19 group compared to control and mild COVID-19 groups. ELISA results for sFas and sFasL are provided in **Figures 3A–D**.

### Correlation analysis of CT scan lung injury and sFas/sFasL protein level in patients with COVID-19

**Figure 4:**
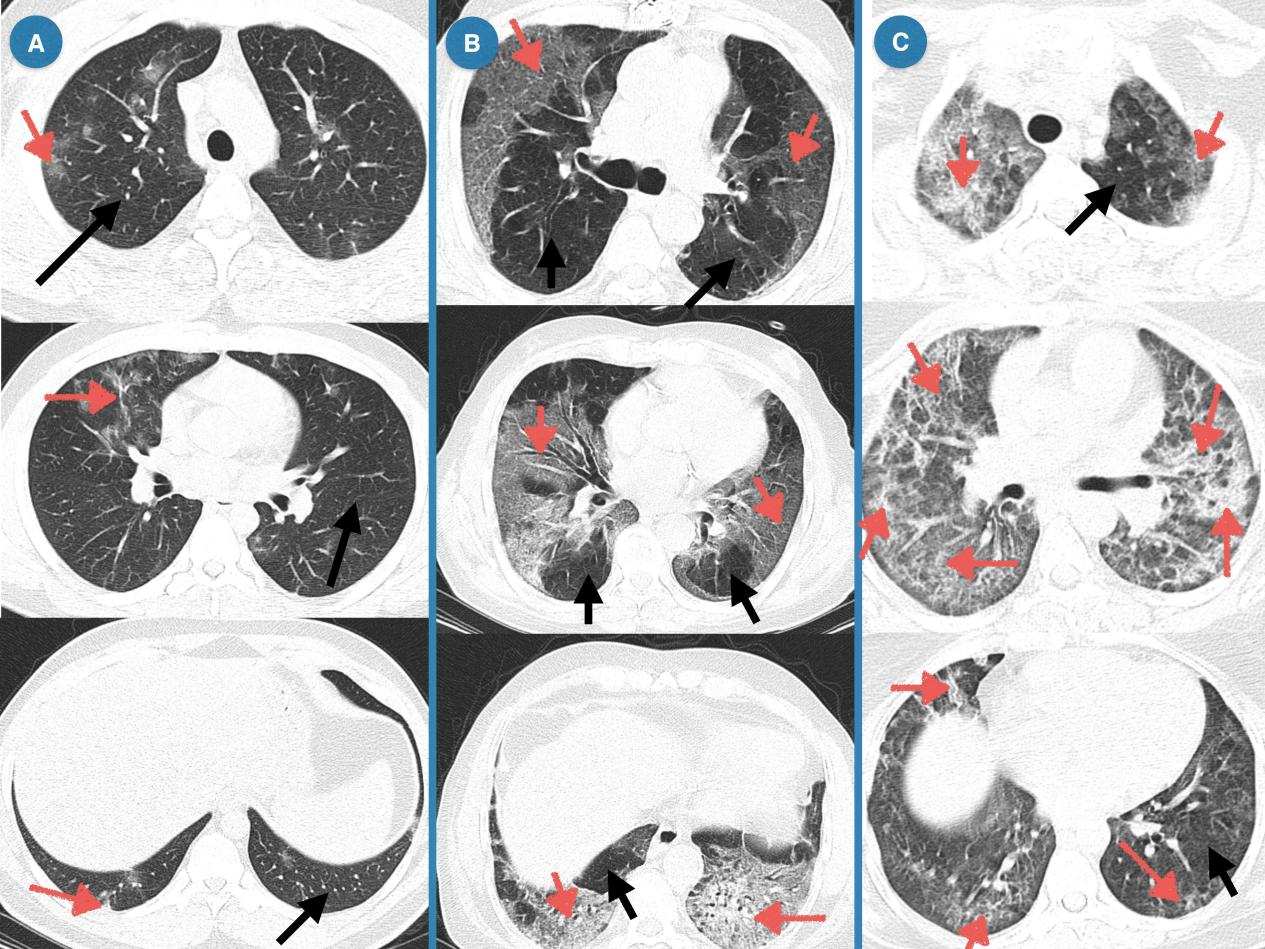
Lung CT of COVID-19 patients representing mild, moderate, and severe respiratory injury. **(A)** Mild, Multiple peripheral and central ground glass opacities (GGOs) in right upper lobe and left lower lobe; Band atelectasis in left lower lobe; **(B)** Moderate, Subpleural GGOs near costal and mediastinal pleura of both lungs, Consolidation with air-bronchogram in lower lobes of both lungs, multiple observations of band atelectasis; **(C)** Severe, Peripheral patchy GGOs, Diffuse and mostly peripheral GGOs, Multiple fibrotic bands.

**Figure 5:**
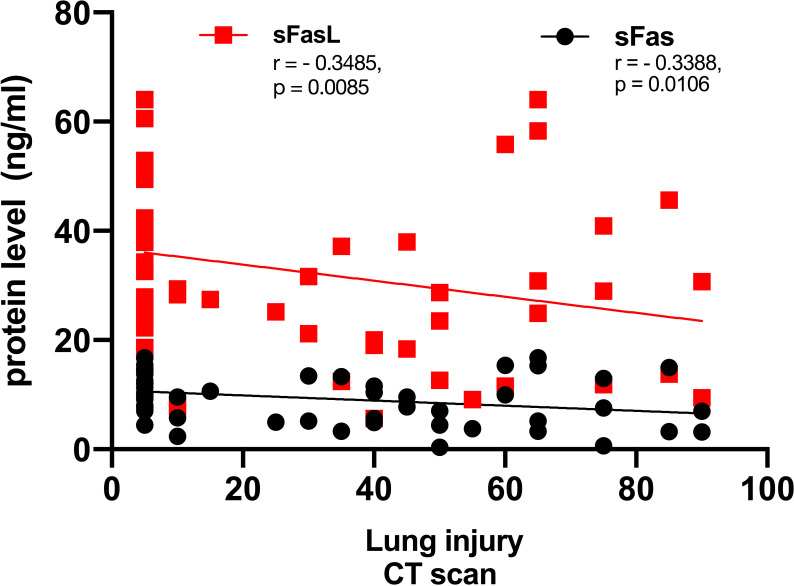
Correlation of lung involvement CT scan and sFas/sFasL in COVID-19 patients. Lung has two right and lobes, that includes upper, middle, and lower lobes. Each lobe is about one-sixth of lung area, and percent of involvement is determined by this method in our department.

In CT-scan, the normal lung appears clear with low attenuation area and normal vascularity, but inflamed/injured areas appear with ground glass opacity (GGO) and abnormal vascularity. Lung CT-scan of COVID-19 cases representing mild, moderate, and severe respiratory involvement are provided in **Figure 4**. Analysis of the lung CT-scan in COVID-19 patients showed that a decrease in sFas and sFasL levels was significantly correlated with severity of respiratory injury (r= - 0.3485, p= 0.0085; r= - 0.3388, p= 0.0106), respectively. These results are indicated in **Figure 5**.

### sFasL protein level predicts mortality in COVID-19 patients

**Figure 6:**
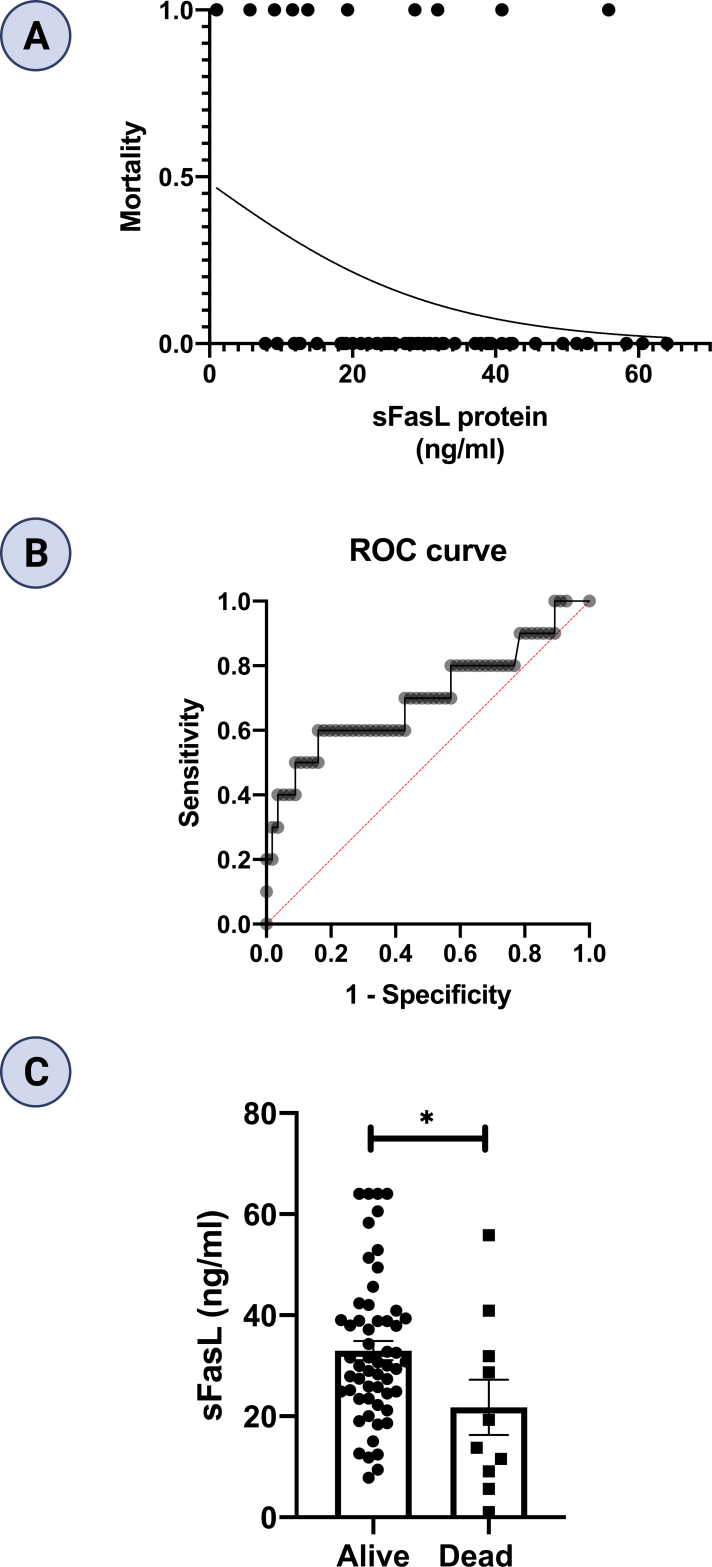
Regression analysis and prognostic performance of sFasL for COVID-19 mortality. **(A)** Regression analysis showed lower sFasL is associated with an increased risk for mortality (n =66). **(B)** AUC in ROC was 0.70. **(C)** sFasL levels were lower in dead compared to alive COVID-19 patients. *p<0.05, **p< 0.01, ***p<0.001, ****p< 0.0001.

In this study, ten patients died, comprising five patients in each of moderate and severe COVID-19 groups. However, none of patients died in the mild COVID-19 group. We performed logistic regression to evaluate the sFas and sFasL levels for prediction of mortality in COVID-19 patients. We found that lower sFasL levels are associated with a significantly increased risk for mortality (OR β1 = 0.940; 95% CI= 0.881-0.991, p = 0.038). Regression analysis and Receiver operating characteristic (ROC) curve are also shown in **Figures 6A, B**. Area under curve (AUC) was 0.70 (p = 0.042). As well, the cut-off value of sFasL was determined 19.65 ng/ml for predicting of COVID-19 patients’ mortality. Finally, we performed statistical analysis to compare the mean sFasL levels in dead vs. alive COVID-19 patients. Our results showed that the mean sFasL levels were significantly (p < 0.05) lower in the dead group compared to the alive group (**Figure 6C**).

### Homology modelling of Fas and FasL structures

In the present study, homology modelling is defined as the prediction of a protein structure (Fas/FasL) based on experimental data. Single template modelling using 4MSV achieved for FasL. Align 2D algorithm showed an alignment score of 123061.55. molpdf and DOPE score are 865.61 and 15026.96. Furthermore, multiple template modelling using 3TJE and 3X3F was achieved for Fas. molpdf score for the best structure is 2547.91. GA341, a MODELLER tool used to rule out bad models, for generated models was higher than 0.6, confirming that the models had good quality.

### Protein structure refinement

**Figure 7:**
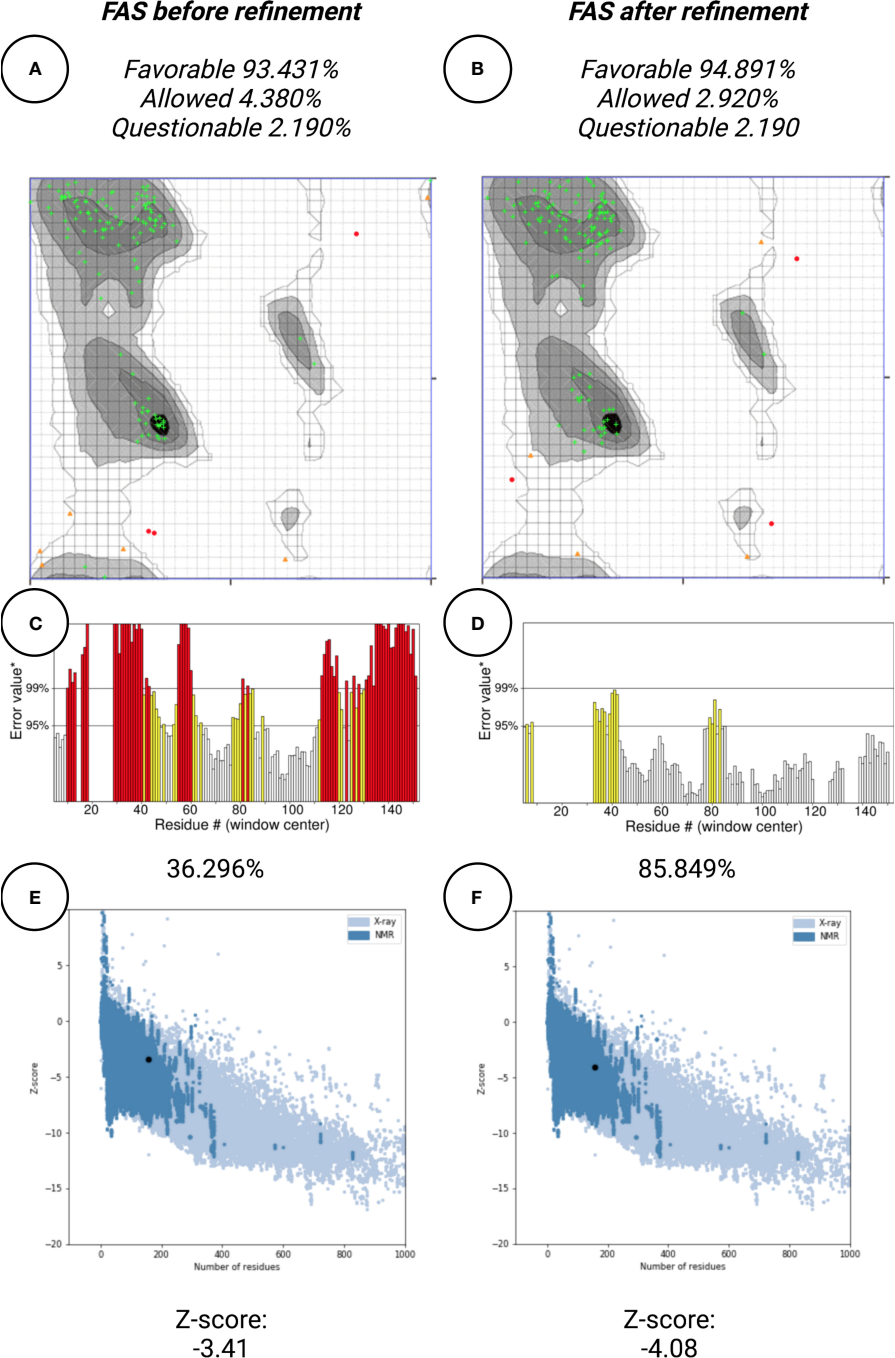
Protein quality verification for Fas tertiary structure. In this illustration, the proteins (Fas/FasL) are evaluated based on structural Bioinformatics rules. After refinement, Fas structures was favorable. **(A, B)** In the Ramachandran plot, more residues are located in favorable regions. **(C, D)** ERRAT score was improved from 36.296% to 85.849%. A score over 80% shows the refined structure has good quality. **(E, F)** ProSA Z-score was improved from -3.41 to -4.08 which is within the acceptable range for structures with the same aminoacid length.

**Figure 8:**
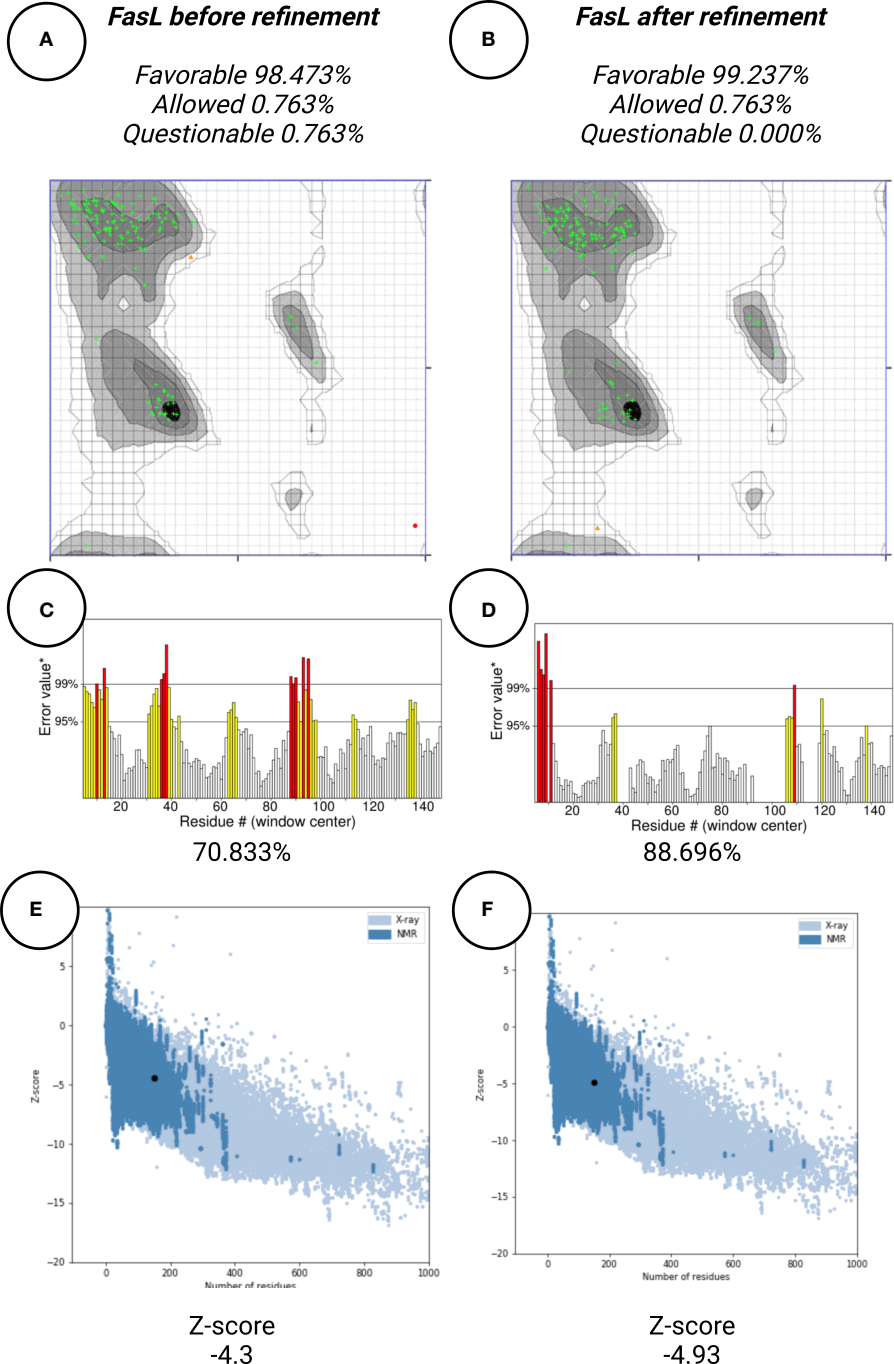
Protein quality verification for FasL tertiary structure. After refinement, FasL structure was favorable **(A, B)** In the Ramachandran plot, more residues are located in favorable regions. **(C, D)** ERRAT score was improved from 70.833% to 88.696%. A score over 80% shows the refined structure has good quality. **(E, F)** ProSA Z-score was improved from -4.3 to -4.93 which is within the acceptable range for structures with the same aminoacid length.

In this work, protein structure refinement is defined as the determination and optimizing of protein structure properties, such as amino acid angles, protein Z-score, alignment of beta-sheets, alpha-helices, and coils. The initial models were refined by GalaxyRefine server, through Refine2 tool set to conservative mode. As shown in **Figures 7A–F** for Fas, RMSD, MolProbity, clash analysis, poor rotamers, Ramachandran preferred, and GALAXY energy for the initial model and the top refined model were 0.000, 3.580, 117.8, 4.9, 90.9, and 1039.44; 8.682, 1.560, 2.7, 1.4, 94.2, and -2838.07, respectively. Also as shown in **Figures 8A–F** for FasL, RMSD, MolProbity, clash analysis, poor rotamers, Ramachandran preferred, and GALAXY energy for the initial model and the top refined model were 0.000, 2.833, 55.3, 4.4, 97.3, and -1184.28; 6.043, 0.887, 1.5, 0.0, 99.3, and -3912.69, respectively. In this step, we refined the Fas and FasL structure.

### Protein structure quality assessment

**Figure 9:**
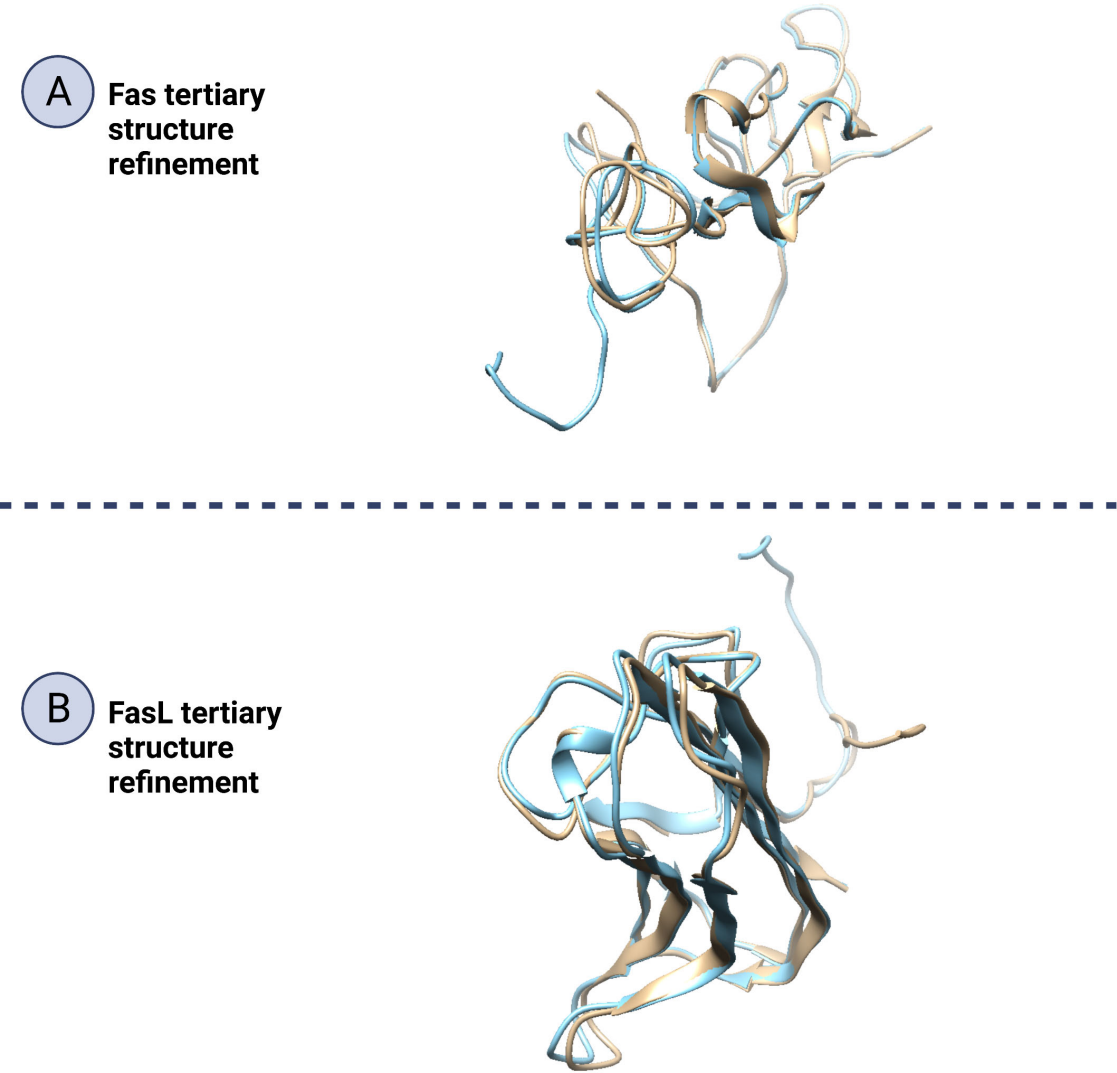
Refinement of Fas and FasL tertiary structure. Protein structure refinement is the determination and improving of a protein’s structural properties. In this figure (**A**; Fas and **B**; FasL), by beige/blue colors, the initial and refined structure have been matched to show how the 3D structures, such as beta-sheets, alpha-helices and coils have been altered to improve the protein quality. Also, a significant portion of the refined structure is made of beta-sheets or alpha-helices indicating good structural stability.

Protein quality assessment is defined as evaluating of quality of the final target protein, based on the previous steps and Bioinformatics analysis rules, for example, the number of amino acids located in most favorable regions should be maximized. For both Fas and FasL, we refined the structures to accomplish a structure that is verified as good quality by various tools, such as Ramachandran plot, ERRAT, and ProSA. For Fas, Ramachandran analysis showed 93.431%, 4.380%, and 2.190% of elements were positioned in the most favored, allowed, and questionable areas. After refinement, mentioned scores were further enhanced, reaching 94.891%, 2.920%, and 2.190%, respectively. For FasL, initial structure showed values of 98.473%, 0.763%, and 0.763%. After refinement, these results were further improved to 99.237%, 0.763%, and 0.000%, respectively. Also, ERRAT scores showed the scores of 36.296% and 85.849% for initial and refined Fas structures, respectively. This analysis showed the scores of 70.833% and 88.696% for initial and refined FasL structures, respectively. These results indicate that the refined structures passed the ERRAT analysis. ProSA analysis of protein Z-score showed values that were within the range of experimentally-verified structures of sequences with the same amino acid length. For Fas, Z-scores for initial and refined structures were -3.41 and -4.08, respectively. Also, for FasL, Z-scores for initial and refined structures were -4.3 and -4.93. These results are shown in **Figures 7**–**9**.

### Docking analysis for Fas/FasL complex

**Figure 10:**
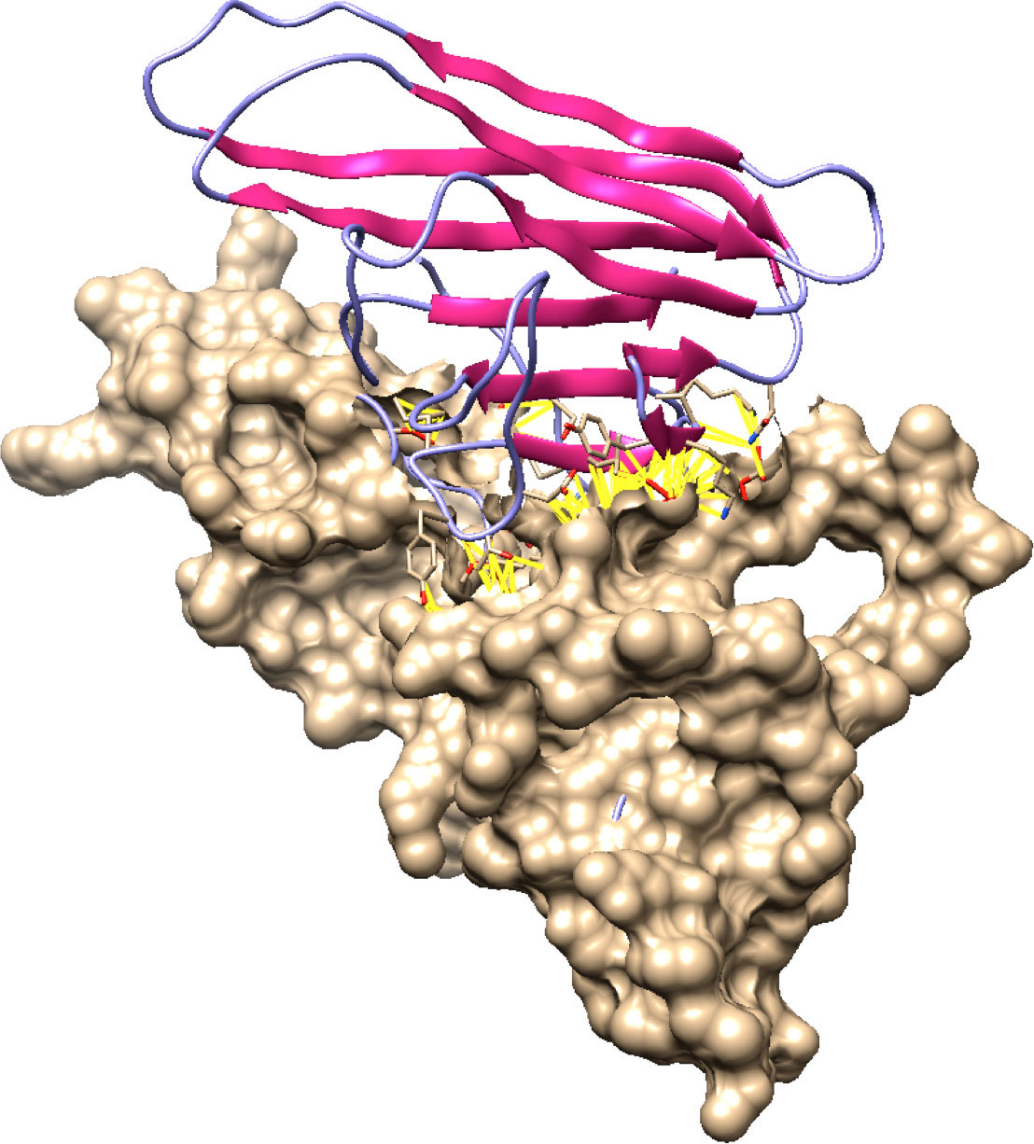
Docked Fas/FasL complex. In this figure, molecular docking has been shown. Docking is the Bioinformatics assessment of how two proteins (Fas and FasL) fit best as a complex. Fas and FasL are shown with distinct colors (purple and beige). Fas/FasL complex contacts are highlighted with yellow lines.

Molecular docking is the evaluation of the way that two or more molecular structures such as protein, enzyme and drugs fit together (34). The final protein structures for best docked complex showed docking score, area, ACE, and transformation of 12864, 1594.40, 137.01, 0.57, and -0.48 -0.04 84.68 -20.55 42.63. After refinement by FireDock, energy (global), attractive VdW, repulsive VdW, ACE, and HB -22.15, -24.02, 4.16, 8.64, and -2.93. The contact residues were detected by UCSF Chimera and default contact criteria. Additionally, 118 contacts were detected in the Fas/FasL complex which are provided in **Table 3** and highlighted in yellow within the docked complex in **Figure 10**.

**Table 3:** Contact area for Fas/FasL complex.

Atom1	Atom2	Overlap	Distance
**LEU 21.A CD2**	SER 43.A CB	1.032	2.728
**ARG 22.A CD**	LYS 46.A CD	0.943	2.817
**LEU 19.A CD1**	LYS 16.A CE	0.934	2.826
**THR 24.A OG1**	ASP 35.A CG	0.917	2.423
**LYS 17.A CD**	GLU 13.A CG	0.887	2.873
**ARG 22.A NH1**	TYR 47.A N	0.826	2.454
**SER 154.A OG**	THR 36.A CG2	0.825	2.515
**SER 156.A CB**	TYR 37.A OH	0.817	2.523
**LYS 17.A CE**	GLU 13.A CG	0.803	2.957
**ARG 22.A N**	LEU 42.A O	0.709	2.351
**LEU 21.A CD2**	SER 43.A OG	0.702	2.638
**ARG 22.A NH1**	TYR 47.A CB	0.667	2.853
**LYS 17.A CG**	GLU 13.A CG	0.667	3.093
**LYS 110.A CE**	PRO 9.A CD	0.642	3.118
**ARG 22.A NE**	LYS 46.A CD	0.629	2.891
**THR 24.A CB**	ASP 35.A CG	0.609	3.151
**THR 24.A OG1**	ASP 35.A OD2	0.595	2.285
**LYS 150.A CE**	PRO 9.A CG	0.591	3.169
**ARG 22.A O**	LEU 42.A CB	0.591	2.709
**LYS 110.A NZ**	PRO 9.A CD	0.573	2.947
**LYS 150.A NZ**	PRO 9.A CG	0.545	2.975
**THR 24.A CB**	ASP 35.A CB	0.543	3.217
**GLU 152.A OE2**	THR 36.A O	0.446	2.394
**ARG 22.A NH1**	TYR 47.A CA	0.443	3.077
**THR 24.A CB**	ASP 35.A OD2	0.441	2.859
**THR 122.A OG1**	ILE 39.A CG2	0.414	2.926
**LYS 17.A CD**	GLU 13.A CD	0.362	3.398
**GLU 152.A CG**	THR 36.A CG2	0.352	3.408
**ARG 22.A NH1**	LEU 42.A CD2	0.335	3.185
**THR 122.A CB**	ILE 39.A CG2	0.323	3.437
**THR 24.A OG1**	ASP 35.A CB	0.303	3.037
**GLU 152.A CD**	THR 36.A O	0.298	3.002
**LYS 110.A NZ**	PRO 9.A CG	0.277	3.243
**LYS 17.A CG**	GLU 13.A CD	0.263	3.497
**LYS 17.A CD**	GLU 13.A OE2	0.23	3.07
**THR 122.A OG1**	ILE 39.A CD1	0.225	3.115
**ARG 22.A CD**	LYS 46.A CA	0.223	3.537
**ARG 22.A O**	LEU 42.A CD2	0.212	3.088
**THR 122.A CB**	ILE 39.A CG1	0.195	3.565
**LEU 21.A CD2**	SER 43.A CA	0.191	3.569
**LEU 19.A CD1**	LYS 16.A NZ	0.178	3.342
**ARG 22.A CG**	LEU 42.A CD2	0.156	3.604
**ARG 22.A CZ**	TYR 47.A N	0.136	3.114
**ARG 22.A NH2**	TYR 47.A O	0.129	2.931
**ARG 22.A O**	LEU 42.A CG	0.12	3.18
**THR 122.A CG2**	ILE 39.A CG2	0.115	3.645
**SER 154.A CB**	THR 36.A CG2	0.111	3.649
**GLU 152.A CB**	THR 36.A CG2	0.096	3.664
**THR 122.A CB**	ILE 39.A CD1	0.082	3.678
**ARG 22.A CB**	LEU 42.A CG	0.054	3.706
**ARG 22.A CB**	LEU 42.A CD2	0.053	3.707
**ARG 22.A CA**	LEU 42.A O	0.048	3.252
**LEU 19.A CD2**	ASN 55.A ND2	0.013	3.507
**LEU 19.A CD1**	GLY 44.A O	-0.008	3.308
**LYS 17.A CB**	GLU 13.A CD	-0.008	3.768
**GLU 152.A CG**	THR 36.A O	-0.008	3.308
**GLU 152.A CG**	THR 36.A CA	-0.01	3.77
**GLU 152.A CD**	THR 36.A CG2	-0.022	3.782
**LEU 21.A CD2**	LEU 14.A CD1	-0.04	3.8
**SER 154.A CB**	THR 36.A OG1	-0.043	3.383
**ARG 22.A CD**	LYS 46.A CE	-0.046	3.806
**THR 24.A CG2**	ASP 35.A OD2	-0.056	3.356
**LEU 21.A CA**	LEU 42.A O	-0.067	3.367
**ARG 22.A CD**	LEU 42.A CD2	-0.072	3.832
**LYS 110.A CE**	PRO 9.A CG	-0.08	3.84
**THR 122.A OG1**	ILE 39.A CG1	-0.093	3.433
**THR 24.A OG1**	ASP 35.A OD1	-0.094	2.974
**LYS 17.A CB**	GLU 13.A OE2	-0.099	3.399
**LEU 19.A CB**	GLY 44.A CA	-0.105	3.865
**SER 154.A OG**	THR 36.A CB	-0.113	3.453
**ARG 22.A CD**	LYS 46.A NZ	-0.114	3.634
**LYS 148.A CE**	PRO 8.A CG	-0.116	3.876
**LYS 17.A CG**	GLU 13.A OE2	-0.119	3.419
**ARG 22.A CD**	VAL 45.A O	-0.141	3.441
**ARG 22.A CD**	LYS 46.A CG	-0.147	3.907
**ARG 22.A O**	LEU 42.A O	-0.157	2.997
**LYS 148.A CD**	SER 6.A O	-0.171	3.471
**ARG 22.A NH1**	LYS 46.A CA	-0.189	3.709
**LYS 148.A NZ**	SER 6.A O	-0.19	3.25
**LEU 21.A C**	LEU 42.A O	-0.191	3.221
**ARG 22.A CD**	LYS 46.A CB	-0.198	3.958
**LEU 19.A CD1**	GLY 44.A CA	-0.206	3.966
**ARG 22.A NH1**	LYS 46.A C	-0.206	3.456
**LYS 17.A NZ**	GLU 13.A CG	-0.207	3.727
**LYS 17.A CB**	GLU 13.A CG	-0.209	3.969
**SER 156.A OG**	TYR 37.A OH	-0.214	3.134
**LYS 17.A CE**	GLU 13.A CB	-0.223	3.983
**ARG 22.A CB**	LEU 42.A O	-0.227	3.527
**ARG 22.A NH1**	TYR 47.A O	-0.238	3.298
**SER 154.A OG**	THR 36.A OG1	-0.247	3.167
**THR 24.A N**	ASP 35.A CB	-0.249	3.769
**ARG 22.A NE**	LYS 46.A CB	-0.251	3.771
**ARG 22.A NE**	LYS 46.A CA	-0.255	3.775
**LEU 21.A CA**	SER 43.A CA	-0.266	4.026
**LYS 17.A CE**	GLU 13.A CD	-0.271	4.031
**THR 24.A O**	THR 36.A OG1	-0.297	3.177
**ARG 22.A N**	LEU 42.A C	-0.299	3.549
**SER 156.A CB**	TYR 37.A CZ	-0.305	3.795
**THR 24.A CA**	ASP 35.A CB	-0.307	4.067
**LYS 150.A NZ**	PRO 9.A CB	-0.32	3.84
**ARG 22.A C**	LEU 42.A CD2	-0.321	3.811
**THR 122.A CB**	ILE 39.A CB	-0.33	4.09
**ARG 22.A CZ**	LYS 46.A CA	-0.345	3.835
**GLU 152.A CG**	THR 36.A CB	-0.35	4.11
**LYS 150.A CE**	PRO 9.A CD	-0.352	4.112
**THR 24.A CG2**	ASP 35.A CG	-0.352	4.112
**LYS 148.A CE**	PRO 8.A CD	-0.353	4.113
**ARG 22.A C**	LEU 42.A CB	-0.356	3.846
**ARG 22.A NE**	LYS 46.A CG	-0.368	3.888
**LEU 21.A CD1**	LEU 14.A CD1	-0.375	4.135
**LEU 19.A CD1**	LYS 16.A CD	-0.377	4.137
**ARG 22.A CZ**	LYS 46.A CD	-0.383	3.873
**ARG 22.A NH1**	TYR 47.A C	-0.393	3.643
**ARG 22.A NH2**	TYR 47.A N	-0.393	3.673
**SER 154.A CB**	THR 36.A CB	-0.395	4.155
**ARG 22.A O**	LEU 42.A CA	-0.396	3.696
**ARG 22.A CG**	LEU 42.A CG	-0.398	4.158

### Molecular dynamics simulation study of Fas/FasL complex for COVID-19 patients

**Figure 11:**
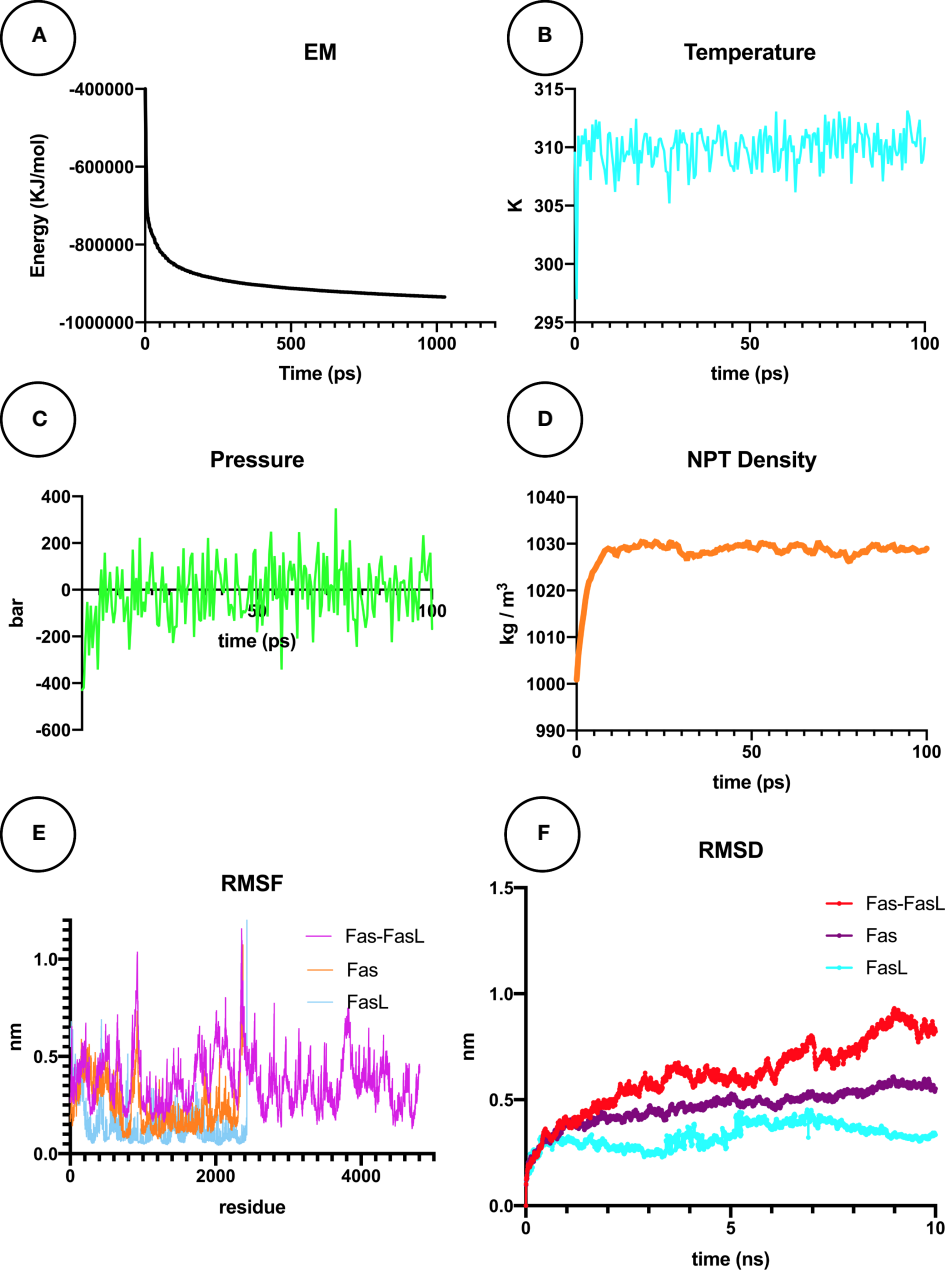
Molecular dynamics simulation (MDS) analyses for Fas/FasL in blood MDS is a simulation approach for analysis of the physical movement of atoms. Here, a biological system of Fas/FasL was virtually set up and equilibrated in blood-like conditions, and was allowed a limited time to evolve. **(A)** Energy minimization (EM) was set-up for the Fas/FasL system. **(B)** During the NVT equilibration, the temperature was matched to blood-like conditions. **(C)** During the NPT equilibration, the pressure reached 0 bar. **(D)** Density of the Fas/FasL system was also equilibrated in this step. After production MDS of the Fas/FasL system: **(E)** Root-mean square fluctuation (RMSF) analysis did not show fluctuation in the Fas/FasL system and the proteins did not denature. **(F)** Root-mean square deviation (RMSD) values shows that the Fas/FasL complex reached stability within the blood-like simulation conditions.

MDS is explained as a computational simulation method that allows the prediction of time-related evolutions of a particular interacting system (35). In our research, the Fas/FasL system was converged to F_max_ < 1000 by the steepest descents algorithm, in 1027 steps, reaching the potential energy of -9.3472875e+05 (**Figure 11A**). After the NVT equilibration, temperature reached 309.782 °K (**Figure 11B**), and after NPT equilibration, the pressure of the system reached 0 bar (**Figure 11C**) and the density was 1027 kg/m3 (**Figure 11D**). For all studied conditions, gyration, RMSF, and visual evaluation of the MDS trajectory did not show denaturation of the protein (**Figure 11E**). RMSD of the Fas, FasL, and Fas-FasL complex stabilized after 5-6 ns (**Figure 11F**). Results for the normal conditions are shown in **Figures 11A–F**.

### Final binding analysis of Fas/FasL interactions in COVID-19

Regarding the PRODIGY analysis, in the control conditions, the average Fas-FasL binding energy was -9.8 kcal.mol^-1^. In the COVID-19 fever condition, the average Fas-FasL binding energy was -8.11 kcal.mol^-1^, showing a stable binding. Also, in COVID-19 hyponatremia condition the binding energy was -10.71 kcal.mol^-1^, which showed a more stable binding state. These results may indicate that a mild increase in temperature can result in slightly less favorable binding while lower Na^+^ levels could enhance binding energies. Nonetheless, binding for all group showed a stable negative ΔG, confirming our experimental data. Binding results for all groups are provided in **Table 4**.

**Table 4:** Binding analysis for molecular dynamics study of Fas/FasL.

Time (ns)	1	2	3	4	5	6	7	8	9	10	Average binding energy (kcal.mol^-1^)
**Normal**	-8.8	-8.9	-8.8	-9.1	-10.2	-9.9	-10.4	-11.0	-10.2	-10.7	-9.8
**Fever**	-7.9	-7.4	-7.2	-7.7	-8.7	-9.2	-8.4	-8.3	-8.4	-7.9	-8.11
**Hyponatremia**	-10.1	-9.8	-9.5	-10.3	-11.6	-11.2	-10.9	-10.8	-11.0	-11.9	-10.71

## Discussion

Several studies have shown that Fas and FasL activate inflammatory cells, particularly neutrophils, through the release of pro-inflammatory cytokines (4, 36, 37). These molecules lead to neutrophils’ and macrophages’ recruitment to injury area (12, 38–41). Uncontrolled inflammation mediated by Fas/FasL plays an immunopathological dysregulated immune response and increased neutrophil counts in various tissue injuries, including respiratory and neurological disorders (12, 42–46). An important adverse effect of hyperinflammation and cell apoptosis in COVID-19 patients is lymphopenia, that is affected by Fas/FasL (47). A recent investigation demonstrated that increased expression of Fas (CD95) in CD4^+^ and CD8^+^ lymphocytes was associated with a reduced proportion of naive events. The findings of their study indicated apoptosis and exhaustion of lymphocytes during COVID‐19 infection (10). Also, in our experiment, moderate and severe COVID-19 groups showed higher mFas expression in comparison with the mild COVID-19 group in PBMCs samples, that consist of lymphocytes, monocytes, and immature neutrophils. Additionally, mFasL expression in PBMCs was higher in COVID-19 group in comparison with the control group. Findings of the present study support our hypothesis that mFas/mFasL is converted to sFas/sFasL. This event in the initial inflammatory phase in the mild COVID-19 patients may be mediated through additional mechanisms such as cleavage by MMPs (12). Further studies are recommended to explore these factors in the inflammatory phase of COVID-19.

Our data confirmed an increase in sFas and sFasL protein production in the initial-stage mild COVID-19 patients. Moreover, we found sFas and sFasL levels were reduced in later-stage more severe COVID-19 groups compared to the mild COVID-19 group. Interestingly, a recent work demonstrated that serum sFasL levels in ICU COVID-19 patients were lower compared to controls at the time of admission (9). Therefore, we suggest that the decrease in sFas/sFasL levels could be due to their consumption in the inflammatory phase of COVID-19 for neutrophil activity and recruitment in particular in the lungs. This phenomenon may indicate the increased activity of proteolytic enzymes such as MMPs or the alternative splicing of full-length Fas mRNA on receptor induction. However, further study is suggested (48).

On the other hand, lymphopenia and neutrophilia are found in patients with COVID-19. A previous study showed that neutrophils release sFasL, that activates apoptosis in some cellular subtypes expressing Fas (49, 50). sFasL promotes inflammatory feedback and overactivation of neutrophils from Type 2 Diabetes Mellitus (T2DM) patients without inducing apoptosis in these cells (4). Another research by Bellesi et al. showed that increased expression of Fas (CD95) in CD4^+^ and CD8^+^ lymphocytes was associated with a reduced proportion of naive events. The findings of their study indicated apoptosis and exhaustion of lymphocytes is induced by Fas during COVID‐19 infection (10). Moreover, next-generation anti-sFasL therapy through inhalation can be used more specifically and directly in future studies. Interestingly, a previous study showed therapy by anti-FasL antibody preserves lymphocytes and virus-exclusive cellular immune feedback, confirming our suggestion (51). According to results of the present work, we think that neutrophilia and lymphopenia in severe-stage COVID-19 patients is related to the role of sFasL.

In addition, it has been demonstrated that Fas/FasL interactions play a role in immune cytotoxicity, affecting peripheral nerve damage and pain (52). Another study found that the concentration of sFasL was significantly associated with the neuropathy severity (53). Findings of the present work indicated that mFas expression has an inverse correlation with myalgia in patients with COVID-19. A recent work showed that serum sFas production is related to a higher risk of nerve injury in Guillain-Barré syndrome (GBS) patients. Also, other studies indicated that myalgia is the most prevalent pain-related symptom after COVID-19–induced GBS (54, 55). Taken together, we believe that Fas/FasL signaling may affect myalgia in COVID-19 cases. Also, analysis of disease severity in COVID-19 patients by lung CT-scan showed that sFas and sFasL proteins are negatively correlated with respiratory injury. Notably, previous research has demonstrated that Fas/FasL pathways and their common genetic variants are related to lung injury and an inflammatory response, which are in line with our findings (56, 57). This is the first evidence supporting a role for sFas and sFasL proteins in respiratory injury in COVID-19 patients.

Strength of this study is that, for the first time, we employed an interdisciplinary approach comprising various clinical findings, laboratory molecular tests, and Bioinformatics simulation (*in silico*) to evaluate Fas/FasL role in COVID-19 patients. We found that sFasL is a prognostic factor for mortality and severity of COVID-19 patients. Another major strength is the confirmation of previous findings regarding sFasL levels in COVID-19 (9) and the potential relation of sFasL levels to lung injury.

A limitation of this research was that it was a single-center study. Therefore, multi-center research with a larger sample size and age-matched participants may be needed to assess these results in COVID-19. Sampling for this study was performed at the beginning of the COVID-19 pandemic. Thus, our study does not include newer variants such as Omicron and delta. We suggest that future research evaluates these results by including other variants of COVID-19. Another restriction of our work was that we could not obtain bronchoalveolar lavage (BAL) fluid. Exploring of sFasL in the BAL fluid in future studies could lead to major advancements. Overall, these limitations do not significantly impact the results of the present study, and our study provides an essential clue for further studies in the absence of possible disruptive factors such as multiple vaccinations. We recommend that future research evaluates the role of sFasL in different variants of COVID-19 to further confirm these results.

COVID-19 presents itself through complicated multi-system symptoms, affecting a wide range of body systems (3). Although different vaccines have been shown to be effective in slowing the COVID-19 pandemic, they have not been adequately successful in providing a permanent immunoprotection. Therefore, pharmacotherapy of COVID-19, in particular by targeting novel inflammation mediators, such as Fas/FasL, should still be studied (8, 58).

## Conclusions

Fas/FasL pathways could play a role in the mediation of hyperinflammatory cytokine response in COVID-19 by being consumed through an interaction with MMPs, and the novel triangle of viral entry, cytokine storm, and multi-system damage. We found the role of Fas/FasL may depend on the disease stage and severity of COVID-19. Our *in silico* analysis confirmed that, alterations in the blood of COVID-19 patients could change Fas/FasL interactions at the molecular level. Preclinical and clinical research testing Fas/FasL-mediating drugs such as anti-sFasL for COVID-19 treatment may aid vaccination efforts to eradicate the COVID-19 pandemic. The findings of the present study indicate that sFasL prognostics severity and mortality in COVID-19 patients. This is the first report describing that the serum sFasL protein is a severity and mortality prognostic marker for the clinical management and therapy of COVID-19 patients.

## Data availability statement

Data are available from corresponding author only on reasonable request.

## Ethics statement

This work was approved by Ethics Committee of Babol University of Medical Sciences, Babol, Iran (IR.MUBABOL.REC.1399.198, IR.MUBABOL.REC.1399.433, IR.MUBABOL.HRI.REC.1399.102). The patients/participants provided their written informed consent to participate in this study.

## Author contributions

AA designed and supervised the study, and prepared the initial and final manuscript. KS and AA conceptualized the study and prepared the initial draft. KS, MS, and MA collected samples. KS, MS, and SM performed the PCR and ELISA experiments. KS performed *in silico* molecular dynamics simulation and docking experiments. AA and KS prepared the final draft. NM and M O critically appraised the manuscript. MJ helped with diagnosis of COVID-1 9 patients. All authors contributed to the article and approved the submitted version.

## Funding

This work was supported by National Elite Foundation of Iran (Grant NO. 1640/151). Also, the present study was funded by the Deputy of Research and Technology (Grant NO. 9909808), Babol University of Medical Sciences, Babol, Iran.

## Acknowledgments

We thank the National Elite Foundation, Mazandaran Province Branch from Iran. We appreciate the assistance of Dr. Ahmad Ghasemi, (MD., Radiologist) in interpreting the lung CT scan of COVID-19 patients in this manuscript. Figures created with BioRender.com.

## Conflict of interest

The authors declare that the research was conducted in the absence of any commercial or financial relationships that could be construed as a potential conflict of interest.

## Publisher’s note

All claims expressed in this article are solely those of the authors and do not necessarily represent those of their affiliated organizations, or those of the publisher, the editors and the reviewers. Any product that may be evaluated in this article, or claim that may be made by its manufacturer, is not guaranteed or endorsed by the publisher.
