# Supplementary material for: Serum soluble Fas ligand is a severity and mortality prognostic marker for COVID-19 patients
Source: Front Immunol. 2022 Aug 31;13:947401. doi: 10.3389/fimmu.2022.947401 (PMC9471328; doi:10.3389/fimmu.2022.947401)
Supplement: Supplementary file 1 [file DataSheet_1.zip › Revised Supplementary/Supp 2.DOCX]

**Supplementary Material – Methods**

STROBE Statement—Checklist of items that should be included in reports of ***case-control studies***

|  | Item No | Recommendation | Explanation |
| --- | --- | --- | --- |
| **Title and abstract** | 1 | (*a*) Indicate the study’s design with a commonly used term in the title or the abstract | The study was indicated as case-control in the methods section. |
|  |  | (*b*) Provide in the abstract an informative and balanced summary of what was done and what was found | Clinical, molecular, and *in silico* findings are described briefly. |
| Introduction | | |  |
| Background/rationale | 2 | Explain the scientific background and rationale for the investigation being reported | We describe the role of hyperinflammation by sFas/sFasL mechanisms in introduction to support the conduct of this study. |
| Objectives | 3 | State specific objectives, including any prespecified hypotheses | The paragraph in introduction briefly discusses the objectives of the present work. |
| Methods | | |  |
| Study design | 4 | Present key elements of study design early in the paper |  |
| Setting | 5 | Describe the setting, locations, and relevant dates, including periods of recruitment, exposure, follow-up, and data collection | Location of patients recruitment, Date range for recruitment of patients. Exposure history (no vaccination, no recent covid-19 infection) and follow-up for mortality by covid-19 are describe in methods. |
| Participants | 6 | (*a*) Give the eligibility criteria, and the sources and methods of case ascertainment and control selection. Give the rationale for the choice of cases and controls | Inclusion and exclusion criteria for covid-19 their severity levels and selection of healthy controls as well as sample size are described. |
|  |  | (*b*) For matched studies, give matching criteria and the number of controls per case | Not applicable |
| Variables | 7 | Clearly define all outcomes, exposures, predictors, potential confounders, and effect modifiers. Give diagnostic criteria, if applicable | Diagnostic criteria of covid-19 has been provided. |
| Data sources/ measurement | 8* | For each variable of interest, give sources of data and details of methods of assessment (measurement). Describe comparability of assessment methods if there is more than one group | Assessment methods are described for each variable. |
| Bias | 9 | Describe any efforts to address potential sources of bias | Analysis was conducted by a double-blind a priori method. |
| Study size | 10 | Explain how the study size was arrived at | We described the sample size. It is in line with similar studies. |
| Quantitative variables | 11 | Explain how quantitative variables were handled in the analyses. If applicable, describe which groupings were chosen and why | Statistical analysis and grouping has been described in detail |
| Statistical methods | 12 | (*a*) Describe all statistical methods, including those used to control for confounding | Done |
|  |  | (*b*) Describe any methods used to examine subgroups and interactions | Done |
|  |  | (*c*) Explain how missing data were addressed | Done |
|  |  | (*d*) If applicable, explain how matching of cases and controls was addressed | NA |
|  |  | (*e*) Describe any sensitivity analyses | We performed ROC analysis and regression for mortality and sFas/sFasL |
| Results | | |  |
| Participants | 13* | (a) Report numbers of individuals at each stage of study—eg numbers potentially eligible, examined for eligibility, confirmed eligible, included in the study, completing follow-up, and analysed | For each stage, number of participants have been clearly provided. |
|  |  | (b) Give reasons for non-participation at each stage | NA |
|  |  | (c) Consider use of a flow diagram | NA |
| Descriptive data | 14* | (a) Give characteristics of study participants (eg demographic, clinical, social) and information on exposures and potential confounders | Demographic data are provided in text and table |
|  |  | (b) Indicate number of participants with missing data for each variable of interest | Data is clearly presented. For example, PCR data is presented with heatmaps. |
| Outcome data | 15* | Report numbers in each exposure category, or summary measures of exposure | Done |
| Main results | 16 | (*a*) Give unadjusted estimates and, if applicable, confounder-adjusted estimates and their precision (eg, 95% confidence interval). Make clear which confounders were adjusted for and why they were included | Done |
|  |  | (*b*) Report category boundaries when continuous variables were categorized | NA |
|  |  | (*c*) If relevant, consider translating estimates of relative risk into absolute risk for a meaningful time period | NA |

| Other analyses | 17 | Report other analyses done—eg analyses of subgroups and interactions, and sensitivity analyses | **Explanation** |
| --- | --- | --- | --- |
| Discussion | | |  |
| Key results | 18 | Summarise key results with reference to study objectives | Done |
| Limitations | 19 | Discuss limitations of the study, taking into account sources of potential bias or imprecision. Discuss both direction and magnitude of any potential bias | Done |
| Interpretation | 20 | Give a cautious overall interpretation of results considering objectives, limitations, multiplicity of analyses, results from similar studies, and other relevant evidence | Done |
| Generalisability | 21 | Discuss the generalisability (external validity) of the study results | NA |
| Other information | | |  |
| Funding | 22 | Give the source of funding and the role of the funders for the present study and, if applicable, for the original study on which the present article is based | Funding information has been provided. |

*Give information separately for cases and controls.

**Note:** An Explanation and Elaboration article discusses each checklist item and gives methodological background and published examples of transparent reporting. The STROBE checklist is best used in conjunction with this article (freely available on the Web sites of PLoS Medicine at http://www.plosmedicine.org/, Annals of Internal Medicine at http://www.annals.org/, and Epidemiology at http://www.epidem.com/). Information on the STROBE Initiative is available at http://www.strobe-statement.org.
